# Genetic assessment wait time indicators in the High Risk Ontario Breast Screening Program

**DOI:** 10.1002/mgg3.359

**Published:** 2018-01-25

**Authors:** Andrea Eisen, Kristina M. Blackmore, Wendy S. Meschino, Derek Muradali, June C. Carroll, Vicky Majpruz, Ellen Warner, Linda Rabeneck, Anna M. Chiarelli

**Affiliations:** ^1^ Medical Oncology Sunnybrook Health Sciences Centre Toronto ON Canada; ^2^ Prevention and Cancer Control Cancer Care Ontario Toronto ON Canada; ^3^ Genetics North York General Hospital Toronto ON Canada; ^4^ Ontario Breast Screening Program Cancer Care Ontario Toronto ON Canada; ^5^ Department of Family Medicine Mount Sinai Hospital Sinai Health System Toronto ON Canada; ^6^ Department of Family and Community Medicine University of Toronto Toronto ON Canada; ^7^ Department of Medicine University of Toronto Toronto ON Canada; ^8^ Dalla Lana School of Public Health University of Toronto Toronto ON Canada

**Keywords:** BRCA1, BRCA2, genetic counseling, genetic predisposition to disease, genetic testing, high risk, magnetic resonance imaging (MRI), mammography, organized breast screening program

## Abstract

**Background:**

The Ontario Breast Screening Program (OBSP) expanded in July 2011 to screen high‐risk women aged 30–69 with annual MRI and mammography. This study evaluated wait time (WT) indicators along the genetic assessment (GA) pathway for women referred to the High Risk OBSP.

**Methods:**

Information was collected for 27,170 women referred to the High Risk OBSP from July 2011 to June 2015 and followed for GA until June 2016. Median duration (days), interquartile range (IQR) were measured for each WT indicator by program year, age, prior breast cancer, and risk criteria.

**Results:**

Among 24,811 women who completed GA, 16,367 (66.0%) had genetic counseling only, 8,444 (34.0%) had counseling and testing and 8,027 (32.4%) met the high risk criteria. Median WT from physician visit to first screen was longer for women having genetic counseling only compared to those having counseling and testing (244 vs. 197 days). Women having counseling only also experienced the longest WT from physician visit to genetic counseling (88 days; IQR = 10–174), which increased by year from 71 to 100 days (*p *< .0001). Among women having counseling and testing, WT from physician visit to counseling was shortest for mutation carriers (39 days; IQR = 4–100). Median WT from testing to laboratory report issue was 41 days (IQR = 22–70) and 17 days to disclosure of test results (IQR = 7–33). Both WTs decreased with year and were shorter for mutation carriers (33 days, IQR = 19–58; 15 days, IQR = 7–28, respectively).

**Conclusions:**

After implementation of the High Risk OBSP, women received timely genetic counseling, in particular those having counseling and testing. Effective triage models for physicians could reduce WT to GA after physician referral.

## 1 INTRODUCTION

Among Canadian women, breast cancer is the leading incident cancer and second leading cause of cancer death (Canadian Cancer Society/National Cancer Institute of Canada, 2016). Women with a family history of breast cancer are at increased risk compared to the general population, with greater risk according to the number and age of affected relative(s) (Bevier, Sundquist, & Hemminki, 2012; Collaborative Group on Hormonal Factors in Breast Cancer, 2001; Pharoah, Day, Duffy, Easton, & Ponder, 1997). About 5% of breast cancer cases are thought to be due to an inherited predisposition due to a mutation in a moderately or highly penetrant cancer susceptibility gene (Claus, Schildkraut, Thompson, & Risch, 1996). The two most common high risk cancer‐predisposing genes are *BRCA1* and *BRCA2*. Although the estimated prevalence in the general population is low (0.11% and 0.12%, respectively) (Peto et al., 1999; Whittemore, Gong, & Itnyre, 1997), carriers have an estimated 40%–87% lifetime risk of developing breast cancer, which typically occurs at a young age (Antoniou et al., 2003; Ford et al., 1998; King, Marks, & Mandell, 2003; Risch et al., 2001). Recent evidence from prospective cohort studies suggests that women at high risk for breast cancer based on their family history or genetic testing, including *BRCA1/2* mutation carriers, benefit from screening that includes magnetic resonance imaging (MRI) in addition to mammography (Kriege et al., 2006; Kuhl et al., 2005, 2010; Leach et al., 2005; Lehman et al., 2005; Sardanelli et al., 2011; Warner et al., 2004, 2011).

The Ontario Breast Screening Program (OBSP) is a province‐wide organized breast cancer screening program offering women aged 50–74 biennial mammograms. Based on recommendations from the Ontario Program in Evidence‐Based Care (Warner et al., 2007, 2012) and the Ontario Health Technology Advisory Committee (Health Quality Ontario, 2010), Cancer Care Ontario engaged an Expert Panel to design a provincial screening program for women at high risk for breast cancer aged 30–69 years. On July 1, 2011 the OBSP expanded its services at 30 screening centers across the province to include annual MRI in addition to digital mammography (DM) for women aged 30–69 years who are at high risk (Chiarelli et al., 2014).

The Expert Panel identified four groups of high risk women who could benefit from MRI and DM screening: women with a deleterious mutation in *BRCA1, BRCA2,* or other gene(s) predisposing to a markedly elevated breast cancer risk; untested first‐degree relatives of a gene mutation carrier; women with a family history consistent with hereditary breast cancer syndrome and estimated personal lifetime breast cancer risk ≥25%; and women who had radiation therapy to the chest (before age 30 and at least 8 years previously). Although the original Ontario guideline on breast MRI for high risk screening (Warner et al., 2007, 2012) did not include women with chest radiation therapy due to the lack of data on MRI screening for this population, the Expert Panel opted to include this group because of their very high breast cancer risk (Travis et al., 2003). Women who meet at least one of the four high risk criteria are also eligible for the High Risk OBSP if they have a history of prior breast cancer and/or other cancers (e.g., ovarian cancer), breast implants, or had a unilateral mastectomy or other breast surgery, as long as they still have palpable breast tissue in at least one breast. Women are ineligible if they have had bilateral mastectomy.

To ensure equitable access for all women in the province, a clinical pathway was developed for the identification, referral, and genetic assessment of women at potential high risk (Cancer Care Ontario, 2015a,b). Criteria for physician referral based on family history were established and two validated models, the International Breast Cancer Intervention Study (IBIS) (Tyrer, Duffy, & Cuzick, 2004) and the Breast and Ovarian Analysis of Disease Incidence of Carrier Estimation Algorithm (BOADICEA) (Antoniou, Pharoah, Smith, & Easton, 2004), were selected for genetic assessment by counselors to identify women at high risk; existing provincial criteria for genetic testing were also implemented. In total 23 genetic assessment centers and 8 laboratories were formally integrated into the High Risk OBSP.

In order to provide access to high‐quality screening, accurate and timely genetic assessment of women at high risk for breast cancer is essential. This study evaluated the physician referral process and wait time indicators across the genetic assessment pathway in the first 4 years of the High Risk OBSP. Wait time indicators were assessed by program year, age group, prior breast cancer history, and risk criteria.

## 2 METHODS

### 2.1 Clinical pathway

**Figure 1:**
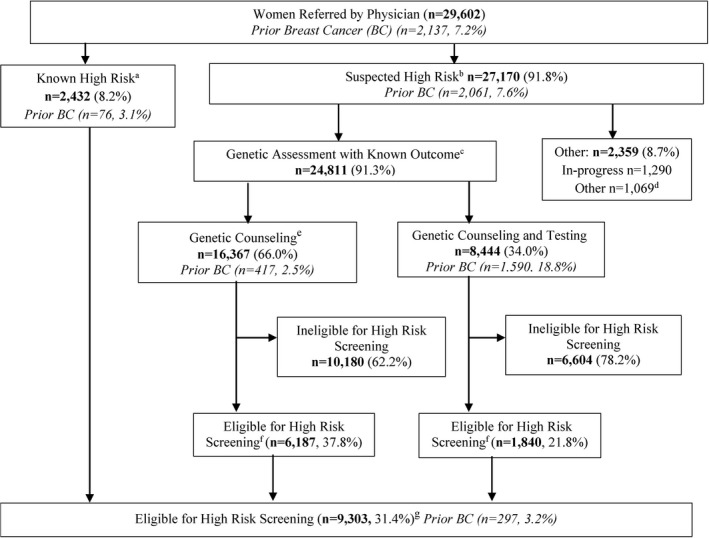
Women referred to the High Risk Ontario Breast Screening Program (July 1, 2011, through June 30, 2015), with follow‐up through June 30, 2016. ^a^Women eligible for direct entry into program if they have at least one of the four high risk criteria; ^b^Women may be eligible and require genetic assessment if they are a first‐degree relative of a carrier of a gene mutation or have a personal or family history of breast or ovarian cancer suggestive of hereditary breast cancer syndrome; ^c^Women who completed genetic counseling only or genetic counseling and testing and who have a known final outcome based on IBIS, BOADICEA and/or genetic testing; ^d^Women who declined genetic counseling only or genetic counseling and testing (*n* = 958) and women who completed genetic counseling only or genetic counseling and testing but final outcome is unknown (*n* = 111); ^e^Among women who had genetic counseling, *n* = 344 were referred for genetic testing but declined; ^f^Women who after genetic counseling only or genetic counseling and testing meet at least one of the four high risk criteria; ^g^Excludes 1,156 women who were never screened because they declined (*n* = 403), deferred (*n* = 282), had bilateral mastectomy (*n* = 146), for other reasons (*n* = 36) or reasons unknown (*n* = 289)

**Figure 2:**
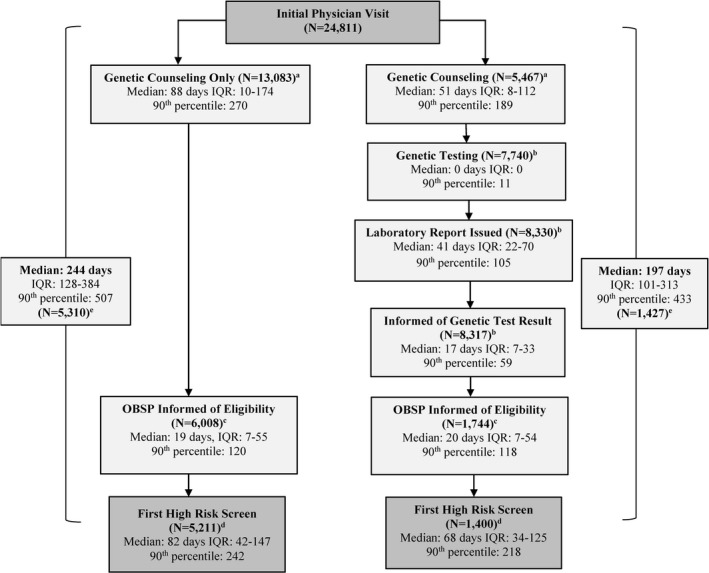
Median durations (days), interquartile ranges (IQR) and corresponding 90th percentiles from initial physician visit date to first high risk MRI (or ultrasound) date among women who completed genetic counseling only or genetic counseling and testing and who have a known final outcome. Abbreviations: IQR, Interquartile Range; OBSP, Ontario Breast Screening Program. ^a^Excludes women who had genetic counseling prior to their initial physician visit date and women who had wait times >365 days; ^b^Excludes women who had genetic testing prior to their counseling date, lab result entered prior to their genetic testing, date they were informed of lab result was entered prior to the laboratory report issued date, and women who had wait times >365 days; ^c^Excludes women where eligibility confirmation date entered prior to genetic counseling date or prior to date informed of genetic test result and women who had wait times >365 days; ^d^Excludes ineligible women, women where first high risk screen date prior to eligibility confirmation date, women never screened and women who had wait times >365 days; ^e^Excludes ineligible women, women where first high risk screen date prior to initial physician visit date, women never screened and women who had wait times >730 days

A clinical pathway outlining patient flow was developed by the OBSP Expert Panel (Cancer Care Ontario, 2015a,b) (Figures 1 and 2). Women are assessed for risk of breast cancer and referred to the program by their physician (primary care or specialist) and fall into one of two categories. If there is prior knowledge that the woman meets at least one of the high risk criteria (“known high risk”) she is automatically enrolled and eligible for screening; this group mainly includes women who were found to be gene mutation carriers prior to 2011, and women with prior therapeutic chest radiation. If the woman is a first‐degree relative of a mutation carrier and has not previously had genetic assessment or has a personal or family history of breast or ovarian cancer suggestive of hereditary breast cancer syndrome, she is referred to a genetic clinic for further risk assessment by a genetic counselor (“suspected high risk”) to determine her eligibility. At the genetic clinic, women may receive genetic counseling only, or they may receive both counseling and testing. All genetic assessment results and screening recommendations are communicated to the High Risk OBSP center and referring physician by the genetic clinic. Women are also informed of their genetic test results by the genetic clinic and may meet with a genetic counselor or geneticist depending on risk. Eligible women found to be mutation carriers and/or assessed as having a ≥25% personal lifetime risk of breast cancer based on the IBIS (Tyrer et al., 2004) or BOADICEA (Antoniou et al., 2004) models are enrolled in the program and arranged for high risk screening by the OBSP. Prior to the first round of screening, women are assessed for any potential contraindications to MRI or reasons why their screening should be delayed. If MRI is contraindicated, the woman is scheduled for a screening breast ultrasound (Kuhl et al., 2010; Sardanelli et al., 2011; Warner et al., 2004). High Risk OBSP navigators are available at each center and facilitate appointments for genetic assessments, screening, and follow‐up of abnormal screens.

### 2.2 Study cohort

The cohort was identified from women referred by their physician (primary care or specialist) to the High Risk OBSP between July 1, 2011 and June 30, 2015 and followed for genetic assessment (genetic counseling with or without testing) to determine eligibility until June 30, 2016. Women who were still in the process of genetic assessment at the time of data extraction, who declined services and for whom results were unknown were excluded.

### 2.3 Data collection

The data used for this study are routine information collected for all women screened within the High Risk OBSP from CCO's Integrated Client Management System database. The *Requisition for High Risk Screening Form*, completed by the referring physician, includes data on date of physician visit, method of referral into the program (direct entry or genetic assessment required), high risk criteria, suggestive history of hereditary breast cancer, and medical history. For women referred for genetic assessment, the *Breast Cancer Genetic Assessment Results Form*, completed by a genetic counselor, collects data on high risk criteria, whether women declined genetic testing, date of genetic counseling and/or testing, date the laboratory report was issued, date the woman was informed of her genetic test results and eligibility for screening. Research ethics approval was not required for this study, because it fell into the category of quality assurance as specified by the University of Toronto Research Ethics Office.

### 2.4 Referral and wait time indicators

To evaluate the physician referral criteria we determined the proportion of women who met one of the four high risk eligibility criteria among women who completed genetic assessment and had a known final outcome. Wait time indicators were developed by the OBSP Expert Panel to ensure timeliness of genetic assessment within an organized setting. Dates of procedures are collected at specific points throughout phases of the program pathway. Wait time indicators examined include: days from initial physician visit to genetic counseling; days from genetic counseling to genetic testing; days from genetic testing to laboratory report issued; days from laboratory report issued to date the woman was informed of her genetic test result; and days from high risk confirmation to first high risk screen (MRI or ultrasound if MRI contraindicated) date.

### 2.5 Statistical analysis

Women having genetic assessment were excluded from this analysis if their genetic counseling date was prior to their physician visit date; their laboratory report result was entered prior to their genetic test date; date they were informed of their genetic test result was entered prior to the laboratory report issued date; or if the wait time exceeded 365 days. Median duration (in days), 90th percentile and interquartile range (IQR) were measured for each wait time indicator. Durations were stratified by program year (2011–2015), age group (30–49 or 50–69 years), prior breast cancer (yes or no) and risk criteria (carriers of a deleterious gene mutation, family history and ≥25% lifetime risk, untested first‐degree relative of a mutation carrier, ineligible for high risk screening). Nonparametric Wilcoxon rank‐sum tests (Haynes, 2013) were performed to compare median wait time for each indicator by year, age, prior breast cancer and risk criteria. All analyses were conducted using SAS version 9.4 (SAS Institute Inc., 2008). A two‐tailed 5% significance level was used for statistical tests.

## 3 RESULTS

Of the 29,602 women aged 30–69 years referred by their physician to the High Risk OBSP, 27,170 (91.8%) underwent genetic assessment to determine their eligibility (Figure 1; “suspected high risk”). Among these women 1,290 were still in process of genetic assessment at the time of data extraction (June 30, 2016), 958 declined services and results were unknown for 111 women. Of the 24,811 women who completed genetic assessment, 16,367 (66.0%) had genetic counseling only and 8,444 (34.0%) had both genetic counseling and testing (Figure 1). Overall, there were 8,027 (32.4%) women considered eligible for high risk screening after completing genetic assessment.

Among eligible women, the overall median wait time from initial physician visit to the first High Risk OBSP screen was longer for women who had counseling only compared to those who had counseling and testing (244 vs. 197 days) (Figure 2). Women who completed genetic counseling only also experienced a longer median wait time to see a counselor after initial physician visit (88 days, IQR = 10–174). Among women having genetic testing, the majority had testing on the same day as counseling and 90% were tested within 11 days of their counseling appointment. The median overall wait time from genetic testing to laboratory report issued date was 41 days (IQR = 22‐70); the wait time for disclosure of genetic test results was an additional 17 days (IQR=7‐33). Median wait time from confirmation of eligibility for the High Risk OBSP until first high risk screen was longer among women who saw a genetic counselor only compared to those who had both counseling and testing (82 vs. 68 days).

The majority of women (64%) who had genetic counseling only were 30–49 years of age and few (3%) had a prior breast cancer (Table 1). Among these women, 96% of those confirmed eligible for the High Risk OBSP had ≥25% lifetime risk based at least in part on family history and 2% were untested first‐degree relative(s) of a mutation carrier. There was a significant increase in median wait time from initial physician visit to genetic counseling in program years 2013–14 (95 days, IQR = 8–173) and 2014–15 (100 days, IQR = 26–189) compared to the first year (71 days, IQR = 3–255; both *p* < .0001) (Table 1). Wait time to genetic counseling was similar for women aged 50–69 and 30–49 years (86 vs. 89 days, *p* = .13), but significantly shorter among women with a prior breast cancer compared to those without (50 vs. 89 days, *p* < .0001). Among risk criteria after genetic counseling, women with a family history and ≥25% lifetime risk waited longer to see a genetic counselor compared to women who were untested first‐degree relative(s) of a mutation carrier (98 vs. 42 days, *p* < .0001).

**Table 1:** Median durations and corresponding 90th percentiles and interquartile ranges (IQR) for time from physician visit to genetic counseling by program year, age group, prior breast cancer, and risk criteria for women who had genetic counseling only (*N* = 16,367)

Physician referral to genetic counseling	Women with known genetic assessment outcome	Included women^a^	Median (days)	IQR (days)	90th Percentile (days)	*p*‐Value
Program year						
2011–2012 (reference)	3,289	2,621	71	3–255	228	—
2012–2013	3,861	3,210	77	5–167	275	.03
2013–2014	4,608	3,618	95	8–173	283	<.0001
2014–2015	4,609	3,634	100	26–189	289	<.0001
Age group^b^						
30–49 years (reference)	10,461	8,349	89	12–174	270	—
50–69 years	5,906	4,734	86	6–173	271	.13
Prior breast cancer						
No (reference)	15,950	12,758	89	11–175	272	—
Yes	417	325	50	0–137	222	<.0001
Risk criteria^c^						
Family history and ≥25% risk^d^ (reference)	5,925	4,606	98	20–186	285	—
1st degree relative of a mutation carrier, declined genetic testing	122	98	42	0–130	189	<.0001
Ineligible for high risk screening	10,180	8,279	85	6–167	262	<.0001

a Excludes women who had genetic counseling prior to their physician visit date (*n* = 2,239) or women who had >365 days between physician visit and genetic counseling dates (*n* = 1,045).

b At time of High Risk OBSP referral.

c If a woman met more than one risk criterion after genetic assessment, the following hierarchy was selected: family history and ≥25% lifetime risk, first‐degree relative of a mutation carrier (but declined genetic testing); known gene mutation carriers were excluded (*n* = 140).

d Based on International Breast Cancer Intervention Study (IBIS) and/or Breast and Ovarian Analysis of Disease Incidence of Carrier Estimation Algorithm (BOADICEA).

Women who had both genetic counseling and testing, were more likely to be 50–69 years of age (55%) and 19% had a prior breast cancer (Table 2). Among these women, 69% of those confirmed eligible for the High Risk OBSP were gene mutation carriers, 29% had a family history and ≥25% lifetime risk and 2% were untested first‐degree relatives of a mutation carrier. Median wait time from initial physician visit to genetic counseling was shorter for program years 2012–13 (48 days, IQR = 3–110) and 2013–14 (44 days, IQR = 6–107) compared to the first program year (57 days, IQR = 14–119, *p* = .002 and *p* = .0009, respectively). It was also shorter for women without a prior breast cancer compared to those with a prior history (49 vs. 62 days, *p* < .0001). Median wait times did not differ between women aged 30–49 and 50–69 years (48 vs. 54 days, *p* = .29). Among risk criteria, the longest wait time to see a counselor was observed for women with a family history and ≥25% lifetime risk compared to gene mutation carriers (78 vs. 39 days, *p* < .0001); wait times were similar between women who were untested first‐degree relatives and mutation carriers (*p* = .11).

**Table 2:** Median durations and corresponding 90th percentiles and interquartile ranges (IQR) for time from physician visit to genetic counseling by program year, age group, prior breast cancer, and risk criteria for women who had genetic counseling and testing (*N* = 8,444)

Physician referral to genetic counseling	Women with known genetic assessment outcome	Included women^a^	Median (days)	IQR (days)	90th Percentile (days)	*p*‐Value
Program year						
2011–2012 (reference)	1,854	1,022	57	14–119	191	—
2012–2013	2,162	1,409	48	3–110	194	.002
2013–2014	2,187	1,496	44	6–107	168	.0009
2014–2015	2,241	1,540	53	10–117	202	.49
Age group^b^						
30–49 years (reference)	3,781	2,506	48	8–109	184	—
50–69 years	4,663	2,961	54	8–113	190	.29
Prior breast cancer						
No (reference)	6,854	4,588	49	7–107	183	—
Yes	1,590	879	62	12–135	208	<.0001
Risk criteria^c^						
Gene mutation carrier (reference)	1,278	764	39	4–100	185	—
Family history and ≥25% risk^d^	535	349	78	22–147	227	<.0001
1st degree relative of a mutation carrier, declined genetic testing	27	22	20	0–70	92	.11
Ineligible for high risk screening	6,604	4,332	51	8–111	183	.002

a Excludes women who had genetic counseling prior to their physician visit date (*n* = 2,781) or women who had >365 days between physician visit and genetic counseling dates (*n* = 196).

b At time of High Risk OBSP referral.

c If a woman met more than one risk criterion after genetic assessment, the following hierarchy was selected: carrier of a deleterious gene mutation, family history and ≥25% lifetime risk, first‐degree relative of a mutation carrier (but declined genetic testing).

d Based on International Breast Cancer Intervention Study (IBIS) and/or Breast and Ovarian Analysis of Disease Incidence of Carrier Estimation Algorithm (BOADICEA).

Median wait time from genetic testing to laboratory report issued date decreased in the third (36 days, IQR = 20–67) and fourth program years (41 days, IQR = 22–70) compared to the first year (43 days, IQR = 25–75; *p* < .0001 and *p* = .0008, respectively) (Table 3). Wait times were longer for women aged 50–69 years compared to 30–49 (45 vs. 36 days, *p* < .0001), those with a prior breast cancer compared to those without (49 vs. 39 days, *p* < .0001), and those with a family history and ≥25% lifetime risk (41 days, IQR = 20–73) compared to mutation carriers (33 days, IQR = 19–58; *p* = .0005).

**Table 3:** Median durations and corresponding 90th percentiles and interquartile ranges (IQR) for time from genetic testing to issue of laboratory report by program year, age group, prior breast cancer, risk criteria for women undergoing genetic testing (*N* = 8,444)

Genetic testing to lab report issued	Women with known genetic testing outcome	Included women^a^	Median (days)	IQR (days)	90th Percentile (days)	*p*‐Value
Program year						
2011–2012 (reference)	1,854	1,818	43	25–75	124	—
2012–2013	2,162	2,144	44	23–71	107	.896
2013–2014	2,187	2,156	36	20–67	105	<.0001
2014–2015	2,241	2,212	41	22–70	94	.0008
Age group^b^						
30–49 years (reference)	3,781	3,726	36	20–63	97	—
50–69 years	4,663	4,604	45	26–76	110	<.0001
Prior breast cancer						
No (reference)	6,854	6,760	39	21–66	102	—
Yes	1,590	1,570	49	30–84	114	<.0001
Risk criteria^c^						
Gene mutation carrier (reference)	1,278	1,256	33	19–58	91	—
Family history and ≥25% risk^d^	535	528	41	20–73	100	.0005
Ineligible for high risk screening	6,604	6,519	43	23–72	107	<.0001

a Excludes women who had laboratory report result entered prior to their genetic testing (*n* = 86) or women who had >365 days between genetic test and laboratory report issued (*n* = 28).

b At time of High Risk OBSP referral.

c If a woman met more than one risk criterion after genetic assessment, the following hierarchy was selected: carrier of a deleterious gene mutation, family history and ≥25% lifetime risk, first‐degree relative of a mutation carrier (but declined genetic testing); women who are a first‐degree relative of a mutation carrier (but declined genetic testing) were excluded (*n* = 27).

d Based on International Breast Cancer Intervention Study (IBIS) and/or Breast and Ovarian Analysis of Disease Incidence of Carrier Estimation Algorithm (BOADICEA).

There was a significant decrease in median wait time from laboratory report issued date to when women were informed of their result in program years 2013–14 (14 days, IQR = 7–27) and 2014–15 (12 days, IQR = 4–26) compared to the first year (22 days, IQR = 9–42; both *p* < .0001) (Table 4). Wait times from laboratory report issued date to disclosure of test results were longer for women aged 50–69 years (18 days, IQR = 8–35) compared to 30–49 (15 days, IQR = 6–29; *p* < .0001) and for women with a prior breast cancer (18 days, IQR = 8–35) compared to those without (16 days, IQR = 7–32; *p* = .0007). Women with a family history and ≥25% lifetime risk had similar wait times to known carriers (14 vs. 15 days, *p* = .223).

**Table 4:** Median durations and corresponding 90th percentiles and interquartile ranges (IQR) for time from issue of laboratory report to date client informed of result by program year, age group, prior breast cancer, risk criteria for women undergoing genetic testing (*N* = 8,444)

Laboratory report issued to client informed of result	Women with known genetic testing outcome	Included women^a^	Median (days)	IQR (days)	90th Percentile (days)	*p*‐Value
Program year						
2011–2012 (reference)	1,854	1,833	22	9–42	76	—
2012–2013	2,162	2,138	21	10–40	70	.472
2013–2014	2,187	2,139	14	7–27	45	<.0001
2014–2015	2,241	2,207	12	4–26	45	<.0001
Age group^b^						
30–49 years (reference)	3,781	3,731	15	6–29	55	—
50–69 years	4,663	4,586	18	8–35	64	<.0001
Prior breast cancer						
No (reference)	6,854	6,749	16	7–32	58	—
Yes	1,590	1,568	18	8–35	64	.0007
Risk criteria^c^						
Gene mutation carrier (reference)	1,278	1,263	15	7–28	55	—
Family history and ≥25% risk^d^	535	523	14	5–32	56	.223
Ineligible for high risk screening	6,604	6,505	17	7–34	60	<.0001

a Excludes women who date they were informed of their result was entered prior to the laboratory report issued date (*n* = 94) or women who had > 365 days between laboratory report issued and date they were informed (*n* = 33).

b At time of High Risk OBSP referral.

c If a woman met more than one risk criterion after genetic assessment, the following hierarchy was selected: carrier of a deleterious gene mutation, family history and ≥25% lifetime risk, first‐degree relative of a mutation carrier (but declined genetic testing); women who are a first‐degree relative of a gene mutation carrier (but declined genetic testing) were excluded (*n* = 27).

d Based on International Breast Cancer Intervention Study (IBIS) and/or Breast and Ovarian Analysis of Disease Incidence of Carrier Estimation Algorithm (BOADICEA).

## 4 DISCUSSION

In the first 4 years of the High Risk OBSP 24,811 women completed genetic assessment, of which one‐third were eligible for high risk screening. The overall median wait time from initial physician visit to high risk screen date was longer for women who had genetic counseling only (244 days) compared to those who had counseling and testing (197 days). The wait time from initial physician visit to genetic counseling was also longer for women referred for counseling only, and this wait time increased with program year. Conversely, wait times from genetic testing to laboratory reporting and disclosure of test results decreased over time. Among high risk criteria, women with a family history and ≥25% lifetime risk who had counseling only experienced the longest wait to see a genetic counselor. Among women having counseling and testing, mutation carriers experienced the shortest wait times along each phase of the genetic assessment pathway.

In the High Risk OBSP 32.4% of women who completed genetic assessment met the high risk criteria. Another study conducted in the United Kingdom (UK) among 22 Regional Genetics Centres assessing familial cancer risk, also found that a low proportion (25%) of referred cases were in the highest risk category (Wonderling et al., 2001). A more recent study conducted in the United States (US) found that among 5,718 women attending a program to identify those at high risk for developing breast cancer, only 15.2% were eligible (Owens, Gallagher, Kincheloe, & Ruetten, 2011). As the process of risk assessment is resource intensive, initial referral to the High Risk OBSP by the physician may require more stringent criteria or a combination of criteria that are more predictive of risk, to reduce the proportion of women referred and assessed unnecessarily. Improved education about program eligibility among referring physicians may also be required.

The longer overall wait time from physician visit to first high risk screen among eligible women having counseling only is expected as these women waited longer to see a counselor and for their first high risk screen. Their wait time along the genetic assessment pathway from initial referring physician visit to genetic counseling also increased by year. This might partly be explained by the increased number of women referred to the High Risk OBSP each year, as the number of genetic assessment centers did not change over time. The delay to see a genetic counselor may also reflect variability in resources and suggests that genetic assessment centers may triage women based on underlying risk, as those women with the shortest wait times (i.e., mutation carriers) would be predicted to be at greatest risk. Developing effective triage models that referring physicians could use to quickly identify patients who are ineligible for the program may help to reduce this wait time. Conversely, decreases in wait times by year from genetic testing to laboratory reporting and to subsequent disclosure of test results, suggests that the current number of laboratories is sufficient to manage the increased volume of women referred each year.

While the wait time to see a genetic counselor after initial physician referral was lengthy in the High Risk OBSP, particularly among women who had counseling only, it is shorter than that reported by others. An earlier study conducted in the UK found that the median wait time from physician referral to counseling among genetic centers assessing familial cancer risk was 19 weeks, ranging from 4–53 weeks across centers due to variation in resources and access inequity (Wonderling et al., 2001). Another survey conducted in the UK showed that some women with breast cancer waited 9 months for their first appointment with a geneticist (Barton, 2006). A randomized quality improvement study among women referred for hereditary breast and ovarian cancer in the US found that 32% of women were seen by a genetic counselor within 3 months from initial physician referral (Rahm, Kulchak, Sukhanova, & Mouchawar, 2007). Our evaluation is more recent and improvements in the genetic counseling process in the past 10 years may have contributed to wait time differences between our study and earlier ones.

In this study wait times along the genetic assessment pathway varied with risk criteria, prior breast cancer history and age. Genetic mutation carriers experienced the shortest wait times along the entire genetic assessment pathway, likely because of the physician referral criteria as these women either had a substantial family history and/or were a member of a family in which a mutation had previously been identified. Among women having counseling only, those with a prior breast cancer may have been seen more urgently by a genetic counselor once referred by their physician if they were newly diagnosed. Conversely, among women with a prior breast cancer who had counseling and testing, the longer wait to see a counselor and from testing to laboratory reporting is unexpected; however, this could be related to the recency of their previous cancer diagnosis, as these women were also slightly older. Younger women experienced shorter wait times from genetic testing to laboratory reporting and were informed of their test results sooner as this information may have influenced treatment decision‐making.

Strengths of this study include its use of routine information collected on a large cohort of women who completed genetic assessment within the High Risk OBSP during a 4 year period. Consequently, we were also able to examine wait time indicators stratified by program year, age group, prior breast cancer and risk criteria. However, it was not possible to distinguish between system‐level and patient‐level factors associated with delays along the genetic assessment pathway. Patients can introduce delay into the testing process by taking days to weeks to return their family history information required to create a pedigree. Provider knowledge (primary care versus specialist) about genetic and other risk factors may also influence the initial referral process.

Although standards for genetic assessment and *BRCA1/2* mutation testing exist (Del Turco et al., 2010; Moyer, 2014), recent studies on clinically significant wait times for genetic counseling and/or testing are lacking. Assessing wait times is important as individuals waiting longer for testing results may experience increases in general distress and anxiety, and a major advantage of genetic testing is reduction in uncertainty regarding one's genetic status (Hilgart, Coles, & Iredale, 2012; Wang, Gonzalez, & Merajver, 2004). The collection of data from the High Risk OBSP allows key performance indicators to be monitored supporting the provision of high‐quality care among women at high risk for breast cancer. One‐third of women who completed genetic assessment in Ontario were eligible for high risk screening and among women having testing 1,278 (15%) were ultimately found to be mutation carriers and experienced the shortest wait times. To improve the initial referral process more stringent criteria are required as well as further education about program eligibility among referring physicians. Effective triage models that referring physicians could use to quickly identify patients who are not eligible would help to increase capacity at centers and reduce wait times to genetic assessment after physician referral.

## CONFLICTS OF INTEREST

The authors declare that they have no conflicts of interest.
